# Different populations and sources of human mesenchymal stem cells (MSC): A comparison of adult and neonatal tissue-derived MSC

**DOI:** 10.1186/1478-811X-9-12

**Published:** 2011-05-14

**Authors:** Ralf Hass, Cornelia Kasper, Stefanie Böhm, Roland Jacobs

**Affiliations:** 1 Laboratory of Biochemistry and Tumor Biology, Gynecology Research Unit, Department of Obstetrics and Gynecology, Medical University, Hannover, Carl-Neuberg-Straße 1, 30625 Hannover, Germany; 2 Institute for Technical Chemistry, Leibniz University Hannover, Callinstrasse 5, 30167 Hannover, Germany; 3 Department of Clinical Immunology and Rheumatology, Medical University, Hannover, Carl-Neuberg-Straße 1, 30625 Hannover, Germany

## Abstract

The mesenchymal stroma harbors an important population of cells that possess stem cell-like characteristics including self renewal and differentiation capacities and can be derived from a variety of different sources. These multipotent mesenchymal stem cells (MSC) can be found in nearly all tissues and are mostly located in perivascular niches. MSC have migratory abilities and can secrete protective factors and act as a primary matrix for tissue regeneration during inflammation, tissue injuries and certain cancers.

These functions underlie the important physiological roles of MSC and underscore a significant potential for the clinical use of distinct populations from the various tissues. MSC derived from different adult (adipose tissue, peripheral blood, bone marrow) and neonatal tissues (particular parts of the placenta and umbilical cord) are therefore compared in this mini-review with respect to their cell biological properties, surface marker expression and proliferative capacities. In addition, several MSC functions including *in vitro *and *in vivo *differentiation capacities within a variety of lineages and immune-modulatory properties are highlighted. Differences in the extracellular milieu such as the presence of interacting neighbouring cell populations, exposure to proteases or a hypoxic microenvironment contribute to functional developments within MSC populations originating from different tissues, and intracellular conditions such as the expression levels of certain micro RNAs can additionally balance MSC function and fate.

## Adult MSC: sources, isolation and culture

**Figure 1:**
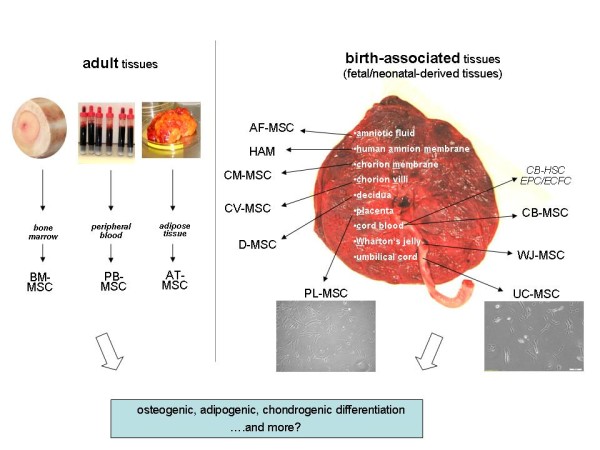
**Major sources of human mesenchymal stem cells (MSC) **. The sources can be distinguished between adult tissues, preferably bone marrow (BM), peripheral blood (PB) and adipose tissue (AT) and neonatal birth-associated tissues including placenta (PL), umbilical cord (UC) and cord blood (CB). Besides cord blood-derived mesenchymal stem cells (CB-MSC) other stem/progenitor cell populations from cord blood also include hematopoietic stem cells CB-HSC and two endothelial populations such as endothelial progenitor cells (EPC) and endothelial colony-forming cells (ECFC).

During the last few years isolations of adult mesenchymal stem cells from different sources have been reported. Bone marrow derived stem cells first described by Friedenstein et al. are still the most frequently investigated cell type and often designated as the gold standard [1]. Mesenchymal stem cells derived from adipose tissue [2], peripheral blood [3], the lung [4] or the heart [5] however have also shown promising potential for proliferation and differentiation into different cell types. In this section we focus on the comparison of adult mesenchymal stem cells derived from bone marrow (BM), adipose tissue (AT) and peripheral blood (PB) (Figure 1).

BM-MSC are isolated from bone marrow aspirate. This invasive procedure is painful for the patient and is accompanied by a risk of infection. The commonly applied preparation method for the generation of MSC from bone marrow is density gradient centrifugation [6]. The collected fraction containing mononuclear cells (MNC) is washed and the cells are seeded on a plastic dish for proliferation. Instead of density gradient centrifugation many groups are using adherence for isolation of MSC from BM.

AT-MSC also termed as adipose-derived stem cells (ASC) are usually isolated from the biological material generated during liposuction, lipoplasty, or lipectomy procedures by enzymatic digestion with collagenase followed by centrifugation and washing [7].

PB-MSC can be obtained from the lymphocyte separation fluid fraction of mononuclear cells after a density gradient centrifugation [3]. Another method is described by Kassis et al. in which PB-MSC are isolated from the mononuclear fraction by loading PB-MSC on fibrin microbeads followed by separation of the cell loaded beads [8].

The amounts of mesenchymal stem cells which can be obtained by these isolations vary enormously. Pittenger et al. isolated MSC from BM by density gradient centrifugation to eliminate unwanted cell types and only 0.001 to 0.01% of the cells isolated from the density interface were mesenchymal stem cells [6]. From 1g of adipose tissue 5 × 10^3 ^stem cells can be isolated, which is 500 times more cells than from an equivalent amount of bone marrow [2,9]. PB-MSC exhibit a colony forming efficiency (CFE) ranging from 1.2 to 13 per million mononuclear cells [10].

Suggested minimal criteria to define human MSC were published in 2006 by Dominici et al.. These suggested criteria included positive expression of CD105 (SH2), CD73 (SH3), CD44 and CD90 and negative expression of CD45, CD34, CD14 or CD11b, CD79α or CD19 and HLA-DR surface molecules. Furthermore, mesenchymal stem cells exhibit plastic-adherence under standard culture conditions and are competent for *in vitro *differentiation into osteoblasts, chondroblasts and adipocytes [11].

## Cell surface marker expression

Several publications demonstrate the reproducible expression of important stem cell markers such as CD44, CD73 (SH3), CD90, CD105 (SH2) and CD166 and the absence of the hematopoietic markers CD14, CD34 and CD45 in BM-MSC and in Wharton's Jelly-derived MSC [6,12]. Schaffler et al. defined the surface marker set for AT-MSC (ASC) as positive CD9, CD29, CD44, CD54, CD73 (SH3), CD90, CD105 (SH2), CD106, CD146, CD166 and HLA I expression and negative CD14, CD31, CD34, CD45, CD133, CD144, HLA-DR, STRO-1 and HLA II expression [13]. De Ugarte et al. performed flow cytometry analyses of BM-MSC and AT-MSC (ASC) and found that both cell types express CD13, CD29, CD44, CD90, CD105 (SH2), CD73 (SH3), and STRO-1. But they found differences in the expression of CD49d, CD54, CD34 and CD106 between the two cell types [14]. Zuk et al. also compared the marker profiles of BM-MSC and AT-MSC (ASC) and found similar expression of CD29, CD44, CD71, CD90, CD105 (SH2) and differences in the expression of CD49d and CD106. AT-MSC (ASC) express CD49d, in contrast to BM-MSC; BM-MSC however, express CD106, which could not be detected in AT-MSC (ASC) [15]. The discrepancies in the expression of STROH-1 and CD34 of BM-MSC and AT-MSC in the different studies may be caused by different isolation methods or different media compositions used which can result in a different expression of surface molecules.

PB-MSC express CD44, CD54, CD105 (SH2) and CD166, but not CD14, CD34, CD45, or CD31 [3]. Kassis et al. isolated PB-MSC which are positive for the expression of CD90 and CD105 (SH2) and negative for CD45 and CD34 [7]. Tondreau et al. performed flow cytometric analyses of BM-MSC and PB-MSC and revealed similar expression patterns, namely the presence of CD44, CD105 (SH2), and CD73 (SH3) and the absence of CD14, CD34, CD45 and HLA-II [16].

The described findings of cell surface marker expression analyses are summarized in table 1.

**Table 1:** 

	BM-MSC	AT-MSC	PB-MSC
positive	CD13, CD44, CD73 (SH3), CD90, CD105 (SH2), CD166, STRO-1	CD9, CD13, CD29, CD44, CD54, CD73 (SH3), CD90, CD105 (SH2), CD106, CD146, CD166, HLA I, STRO-1	CD44, CD54, CD90, CD105 (SH2), CD166

negative	CD14, CD34, CD45	CD11b, CD14, CD19, CD31, CD34, CD45, CD79α, CD133, CD144, HLA-DR	CD14, CD34, CD45, CD31

Reference	[6,14]	[13,15]	[3,7,16]

## MSC derived from birth-associated tissues

In addition to distinct adult tissues (adipose tissue, bone marrow, peripheral blood), MSC can be obtained from several birth-associated tissues including placenta, amnion, umbilical cord (UC) and cord blood (CB) (Figure 1). A significant advantage of these neonatal tissues is their ready availability, thus avoiding invasive procedures and ethical problems. Moreover, birth-associated tissues harbor a variety of embryonic or premature cell populations including MSC, endothelial stem/progenitor cells (EPC, ECFC) and hematopoietic stem cells (CD34^+^, CD133^+^). It is also suggested that MSC from these neo-natal tissues may have additional capacities in comparison to MSC derived from adult sources. Indeed, several studies have reported superior cell biological properties such as improved proliferative capacity, life span and differentiation potential of MSC from birth-associated tissues over BM-MSC.

For example, MSC from the human placenta (PL-MSC) have been reported to have a higher expansion and engraftment capacity than BM-MSC [17-19]. In this context, it is important to note that placental tissue can be fetal or maternal in origin requiring the two types of tissue to be individually characterized with respect to MSC function. According to the first international workshop on placenta-derived stem cells, four regions of fetal placenta can be discriminated: amniotic epithelial, amniotic mesenchymal, chorionic mesenchymal, and chorionic trophoblastic tissue. Consequently, at least four different cell populations with stem or progenitor properties can be distinguished: human amniotic epithelial cells (hAEC), human amniotic mesenchymal stromal cells (hAMSC), human chorionic mesenchymal stromal cells (hCMSC), and human chorionic trophoblastic cells (hCTC) [20]. Placenta-derived MSC from fetal tissue, including amnion membrane ((AM-MSC) (or HAM; human amniotic membrane)) [21-24], chorion membrane (CM-MSC) [25,26] and chorionic villi (CV-MSC) [27-29] have been have been described as having a more limited life span than MSC populations obtained from the maternal part of the extraembryonic membranes or decidua (D-MSC) [25,26,30] (Figure 1). Moreover, clonal subpopulations of D-MSC have been attributed with the potential to differentiate into tissues from all three germ layers [31]. A similarly high cellular plasticity for differentiation into the three germ layers has also been described for MSC derived from amniotic fluid (AF-MSC) [32], amniotic epithelial cells [22] and endometrial regenerative cells [33].

A certain heterogeneity within the stromal or stem cell population displaying mesenchymal-like characteristics such as surface marker expression, plastic adherence, self renewal and differentiation capacity has also been identified in MSC derived from the umbilical cord (UC-MSC). Separation of UC-MSC by counterflow centrifugal elutriation resulted in differentially sized subpopulations displaying altered proliferation potentials which were associated with significantly different amounts of senescent cells [34].

With respect to MSC isolation from umbilical cord, the different parts of this tissue should also be considered individually [35,36]. MSC can be isolated from whole umbilical cord [37], from Wharton's jelly (WJ-MSC) [38-40] or from umbilical cord blood (CB-MSC), [41,42] which also harbors hematopoietic stem cells, endothelial precursor cells and endothelial colony-forming cells (Figure 1). Proteome analysis of WJ-MSC revealed differences in the protein expression pattern during in vitro self-renewal [43] and other work has demonstrated that UC-MSC represent a preferred population for musculoskeletal tissue engineering [44]. Like PL-MSC and other neonatal birth-associated MSC, the UC-MSC exhibit certain cell biological properties which are different from MSC originating from adult sources (AT-MSC (ASC), BM-MSC, PB-MSC).

## Comparison of the proliferation capacity between AT-MSC (ASC) and UC-MSC

The proliferation capacity and senescence of these cells have been analyzed by many scientists over the last few years. The proliferation capacity of cells is important with regard to their application in cell therapy and tissue engineering. Baksh et al compared umbilical cord perivascular cells (UCPVC) to BM-MSC and determined that the UCPVCs also have a higher proliferation capacity than the BM-MSC [45]. Moreover, various papers have been published demonstrating that UC-MSC exhibit a higher proliferation capacity than BM-MSC [46-49]. Lu et al. performed proliferation studies with BM-MSC and UC-MSC which revealed that BM-MSC showed significantly slower population doubling times. The mean doubling time of the UC-MSC in passage 1 (P1) was about 24h and remained almost constant up to P10. In contrast the mean doubling time of BM-MSC was 40h and increased considerably after P6 [49]. They determined the population doublings over 20 days and observed that after three days both cell types showed similar population doubling times, but that from day seven on the population doubling time of the UCPVCs was significantly increased [46]. Additionally, they found that the UCPVCs continued to grow by multi-layering, in contrast to the proliferation of BM-MSC that was inhibited due to contact inhibition. AT-MSC (ASC) have also been shown to have higher proliferation capacities than BM-MSC [50]. Peng et al. described population doubling times of 45.2 h for AT-MSC (ASC) and 61.2 h for BM-MSC. Moreover, they revealed that the BM-MSC were morphologically larger as compared to AT-MSC (ASC) [51]. It should however be noted that differences in the doubling times of AT-MSC (ASC) originating from different regions of the body have been reported [52,53]. Van Harmelen et al. published that AT-MSC (ASC) from the subcutaneous adipose tissue region proliferated faster (doubling time, 4 +/- 1 days) than those from the omental region (doubling time, 5 +/- 1 days) [54]. In addition to the origin of the cells, the cultivation conditions and various medium supplements may have an effect on doubling times of the AT-MSC (ASC). Own experiments revealed shorter doubling times for AT-MSC (ASC) cultured in human serum instead of fetal calf serum (unpublished data).

Besides the higher proliferative activity of UC-MSC the cells show no sign of senescence over several passages [55,56]. Conconi et al. cultured UC-MSC over 16 serial passages and found no variation in cell morphology or senescence [57]. Mitchell et al. cultured porcine UC-MSC for more than 80 doubling times with no decrease of proliferative capacity [58]. Kern et al. investigated the senescence ratio of AT-MSC (ASC) in comparison to BM-MSC. AT-MSC (ASC) could be cultivated up to passage number 8 without any sign of senescence whereas in BM-MSC senescence was demonstrated already in cells from passage number 7 [50].

## Differentiation capacity and plasticity of AT-MSC (ASC) and UC-MSC

The differentiation of UC-MSC and AT-MSC (ASC) along the adipogenic, chondrogenic and osteogenic lineages has been investigated by many working groups. Furthermore, *in vitro *differentiation into cardiomyocytes [40,59], endothelial cells [48,60] or neuronal cells [61,62] has been reported.

### Adipogenic differentiation

Adipogenic differentiation is usually defined by the appearance of cells containing intracellular lipid droplets. Both AT-MSC (ASC) and UC-MSC have been successfully differentiated into adipocytes [7,63]. For preadipocyte differentiation of AT-MSC (ASC) a high cellular density and a subsequent growth arrest at the G0/G1 boundary are important [64,65]. Furthermore FGF2, thiazolidinediones like troglitazone, pioglitazone, rosiglitazone and 17-β estradiol have been shown to induce adipogenic differentiation of AT-MSC (ASC) [66-68]. Hu et al successfully differentiated UC-MSC into adiopocytes by medium supplementation using dexamethasone and insulin [69]. Oil red staining is commonly applied to verify adipogenic differentiation.

### Chondrogenic differentiation

The chondrogenic differentiation capacity of MSC is evidenced by the formation of shiny cell-spheres expressing type II collagen in pellet cultures. Chondrogenic differentiation of AT-MSC (ASC) and UC-MSC has been described by many groups using medium supplements such as transforming growth factor β1, ascorbate-2-phosphate, and dexamethasone [69,70]. Feng et al. promoted chondrogenic differentiation of AT-MSC (ASC) by the addition of growth and differentiation factor-5 (GDF5) [71] and stimulation by FGF-2 or BMP-6 has also been reported [72,73]. Successful chondrogenic differentiation is indicated by the detection of the extracellular matrix component glycosaminoglycan (GAG), by immunohistological staining e.g. of collagen II and aggrecan or by verification of the expression of typical genes of the chondrogenic lineage via PCR.

### Osteogenic differentiation

Enhanced alkaline phosphatase expression and mineralization assayed by von Kossa or alizarin red staining indicates the occurrence of osteogenic differentiation. Different groups reported differentiation protocols for AT-MSC (ASC) by using dexamethasone, β-glycerophosphate and ascorbic acid as medium supplements [50,74,75]. The identical medium composition was used for the successful osteogenic differentiation of UC-MSC [69]. Medium supplementation by 1,25-dihydroxyvitamin D3 [76,77] or BMPs [69,78,79] has also been reported to enhance osteogenic differentiation.

## Effects of oxidative stress and hypoxia in MSC

**Figure 2:**
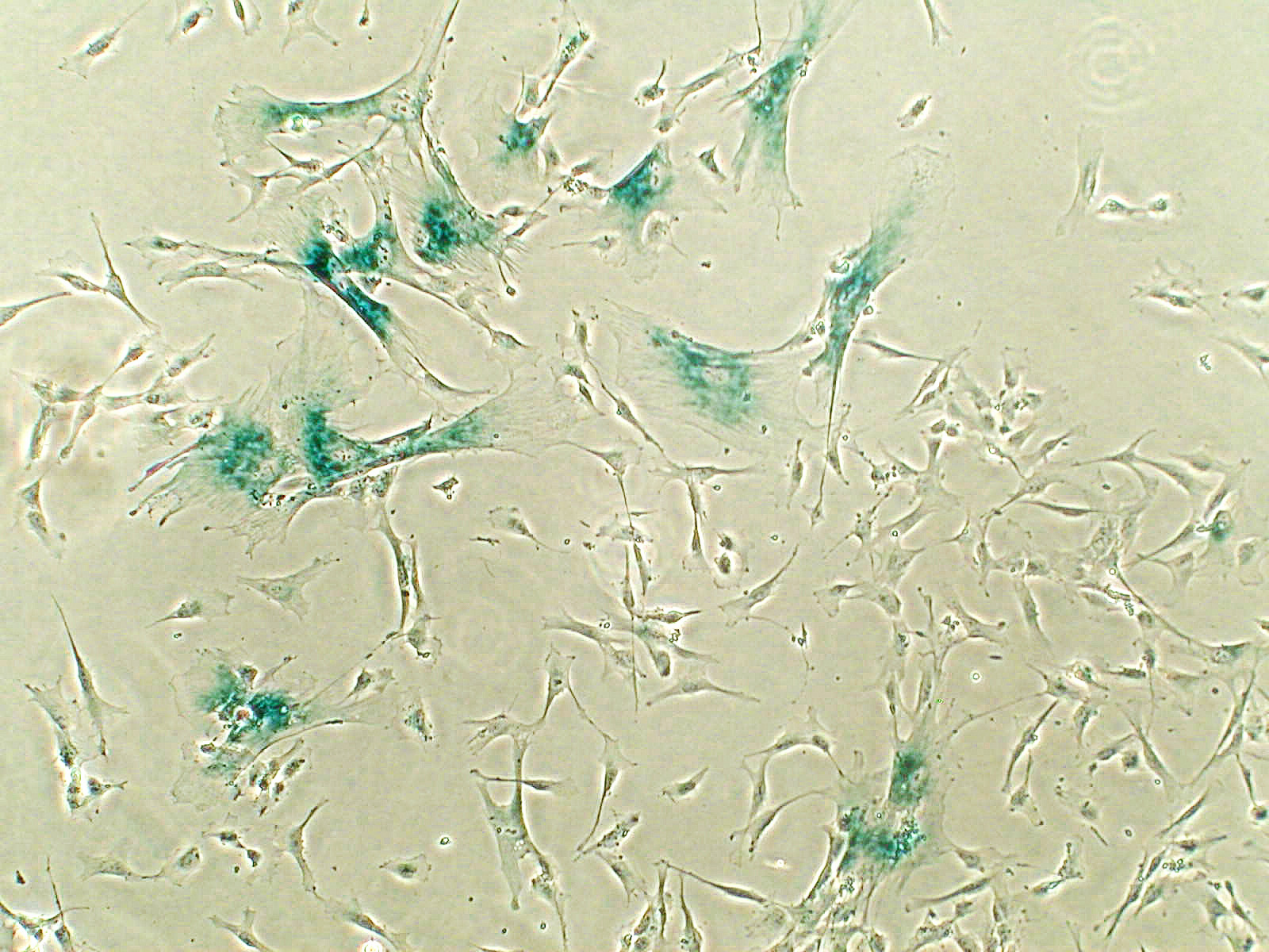
**Senescence-associated β-galactosidase (SA-β-gal)-positive cells in the UC-derived mesenchymal stem cell culture under low (2%) serum concentration **. Nuclei of senescent cells are surrounded by cyan dye. Moreover, serum deprivation is accompanied by a decrease of the proliferative potential in the cells and a significant increase in cell size.

Differences in cell functions between MSC populations derived from adult or neonatal tissues are also influenced by the microenvironment. Within the appropriate tissues *in vivo*, stem cells like MSC are usually present in stem cell niches under hypoxic conditions. Therefore, *in vitro *primary culture in a normoxic atmosphere (21% O_2_) can be considered as an exposure to enhanced oxidative stress and promotes the generation of metabolic radicals or reactive oxygen species (ROS). The intracellular accumulation of ROS can cause protein and DNA damage if these compounds are insufficiently metabolized by an appropriate anti-oxidative defense system. Consequently, ROS accumulation at high oxygen levels induces elevated apoptosis and premature aging by STASIS (stress or aberrant signaling-inducing senescence) [80]. Indeed, MSC cultured under normoxic conditions exhibit premature senescence and a reduction in population doublings in comparison to cells cultured under hypoxia [81,82] and may also show restricted cell division due to telomere shortening and replicative senescence [80,83]. The migratory capability of MSC cultured under hypoxic conditions is also enhanced in contrast to that seen in normoxia [84]. Hypoxic conditions therefore influence proliferation and cell fate commitment, meaning that gradients of oxygen tensions influence the prolonged maintenance of a stem cell phenotype and pluripotency [85]. Moreover, serum starvation and deprivation of growth factors can promote premature aging in MSC (Figure 2) and studies of MSC in a hypoxic environment show that serum starvation can be associated with massive cell death [86].

Previous work has demonstrated that the culture of MSC under hypoxic conditions is accompanied by increased Oct4 expression and telomerase activity [81] which are involved in the maintenance of stemness. Other studies have identified changes in the transcriptome of hypoxic stem cells that seem to indicated that hypoxic conditions rather reflect a physiologically normoxic environment for the cells [87].

Hypoxic conditions induce the transcription factor hypoxia-inducing factor-α which can promote certain differentiation phenotypes in MSC. Thus, chondrogenic differentiation of AT-MSC (ASC) has been observed at enhanced levels under hypoxic conditions where osteogenesis is inhibited. In contrast, enhanced osteogenic differentiation of AT-MSC (ASC) can be induced under normoxia [88,89]. Moreover, other MSC populations including UC-MSC exhibit differences in energy turnover and the expression of energy metabolism-associated genes at different hypoxic conditions [90]. Functional changes of MSC under hypoxia also include increased secretory activity, i.e. of vascular endothelial growth factor and interleukin-6 as well as mobilization and homing by the induction of stromal cell-derived factor-1 expression and the corresponding receptor CXCR4 [91]. In this context, MSC subpopulations displaying a high aldehyde-dehydrogenase activity have been reported with increased responsiveness to hypoxia, including an upregulation of Flt-1, CXCR4 and angiopoietin-2 [92].

Together, these findings further substantiate that oxygen tensions contribute to the regulation of MSC function and fate. Whereas MSC can display and maintain a hypoxic microenvironment within the appropriate tissues *in vivo*, these conditions may also contribute to the control of other important cellular functions of MSC including their immune-modulatory properties.

## MSC immune function

**Figure 3:**
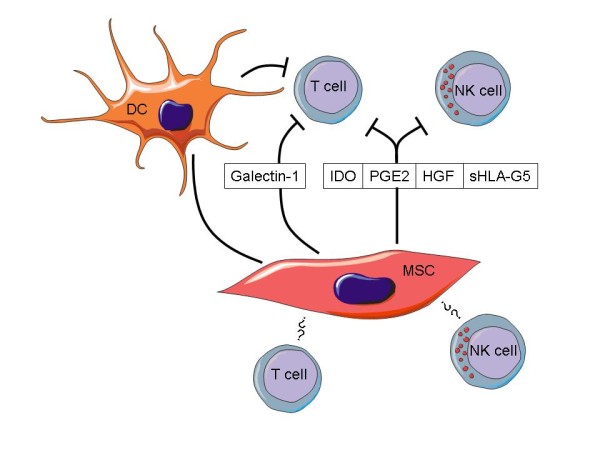
**MSC mediate immunosuppression of T and NK cells via different mechanisms **. Soluble factors secreted by MSC such as iNOS, IDO, PGE2, sHLA-G5 can suppress T- and NK cell functions, whereas galectin-1 inhibits T cells but not NK cells. In addition, MSC can indirectly mediate immunosuppression by inhibiting dendritic cells and inducing the expansion of regulatory T cells (Tregs). Furthermore, MSC can directly interact with T and NK cells via cell to cell contact. However, receptors and ligands involved in the cell contact-dependent interaction mechanisms are still largely unknown.

Two outstanding features of MSC are relevant to immunity: 1) immunosuppression and 2) the so called immunoprivilege. What do these terms mean? MSC-mediated immunosuppression describes the fact that MSC are able to suppress several functions exerted by diverse immunocytes such as T-, B-, and NK cells. The affected functions comprise proliferation, production of soluble factors (e.g. cytokines), and cellular cytotoxicity (Figure 3). Immunoprivilege means that MSC themselves are somehow protected from immunological defence mechanisms. Undoubtedly, there is much truth in the reports of MSC-mediated immune effects. Nevertheless, there also seems to be some conflicting data. The inconsistencies between some reports may, however, be due to the population diversity of the primary cultures and to the tissue- and species-origin of the MSC tested. As mentioned above, MSC are currently characterized using a minimum of surface markers, which might not be sufficient for their precise definition. A further cause for conflicting data could be the source (adipose tissue, blood, bone marrow, umbilical cord, umbilical cord blood) for isolation of the MSC. In order to review the immunological findings in MSC research we screened the literature and compared the data on immunological interactions between MSC and immune cells.

### Species-related variations of MSC-mediated immune modulation

Immunosuppressive effects of MSC have been reported for all species tested so far. In mice, Interferon (IFN)-γ activation of MSC was shown to be effective in treatment and prevention of graft versus host disease (GvHD) [93]. The infusion of MSC in combination with rapamycin reduced alloimmune responses and promoted tolerance in a cardiac allograft mouse model [94]. Pretransplant infusion of MSC in mice prolonged graft survival in semiallogeneic heart transplantation [95]. In rats, long term acceptance of solid organ allografts has been observed in animals treated with MSC in combination with low-dose mycophenolate [96]. Baboon MSC suppressed lymphocyte proliferation in vitro and prolonged skin graft survival in vivo [97]. Le Blanc et al demonstrated that human MSC can inhibit proliferation of lymphocytes, APCs and NK cells in mixed lymphocyte reactions (MLR) [98]. In a recent phase II study, GvHD in humans could be ameliorated upon hematopoietic stem cell transplantation (HSCT) [99] and it was found that MSC expand in vivo irrespective of the donor as MSC from HLA-identical sibling donors, haploidentical donors, and third-party HLA-mismatched donors were equally effective. Although most of the studies revealed MSC to be immunosuppressive, Nauta et al found in mice that donor-derived MSC are immunogenic in an allogeneic host and stimulate donor graft rejection in a nonmyeloablative setting [100]. Indoleamine 2,3-dioxygenase (IDO) and inducible nitric oxide synthase (iNOS) are substances involved in MSC mediated immunosuppression. However, when MSC were examined after stimulation with their respective inflammatory cytokines in human and mouse, human MSC were found to express extremely high levels of IDO, and very low levels of iNOS, whereas mouse MSC expressed abundant iNOS and very little IDO, further underscoring the observed species variations in MSC-mediated immunosuppression [101].

### Differences in the immunosuppressive capacity of MSC from different tissues

MSC characteristics do not only depend on species specific factors but also on the tissue source from which they were harvested. MSC can be isolated from many tissues including bone marrow (BM), adipose tissue (AT), placenta (PL), umbilical cord (UC, Wharton's jelly) or umbilical cord blood (UCB), respectively. Most experiments in mice, rats and monkeys were performed using BM-MSC. In the human system, BM-MSC are used most frequently, but AT, PL and especially UCB also serve as sources for MSC isolation. In case of UCB-MSC, the easy and risk-free availability of UCB is negatively counterbalanced by the lower yields of MSC from this source [102]. In contrast, the umbilical cord tissue or Wharton's jelly is an excellent source for isolating MSC [103-105]. Source-related features of MSC might directly contribute to the diversity of opinions regarding the mechanisms (soluble factors versus cell-to-cell contact) of MSC-mediated immunomodulation. It is still a matter of debate if the regulatory effects are cell-to-cell contact-dependent, or if, as postulated by most groups, soluble factors are sufficient [106]. The contact dependency of MSC-mediated effects has been much less investigated than the soluble factors effective in immunosuppression. In most of these studies transwell systems were used, and showed indirectly that cell-to-cell contact is required as MSC-mediated effects were abolished or diminished when effector and target cells were separated by a membrane. The molecules involved in the cross talk however remained largely obscure in most reports. In our experiments using UC-MSC we found that NK cell suppression such as decreased proliferative and cytotoxic capacity strictly requires cell-to-cell contact (unpublished data).

### Soluble factors mediating MSC-dependent immune regulation

What are the mechanisms enabling MSC to regulate functions of immunocytes? As can be envisaged from the diversity of the results reported from different groups there is, as yet, no clear answer. However, several factors that contribute to the MSC-mediated effects have been identified. MSC constitutively or upon stimulation secrete large amounts of soluble factors such as interleukin(IL)-1, IL-6, IL-8, IL-7, IL-8, IL-10, IL-11, IL-12, IL-14, IL-15, leukemia inhibitory factor (LIF), granulocyte colony-stimulating factor (G-CSF), granulocyte macrophage colony-stimulating factor (GM-CSF), stem cell factor (SCF), macrophage colony-stimulating factor (M-CSF) fms-like tyrosine kinase-3 ligand (flk-3L), CCL2, tissue inhibitor of metalloproteinase (TIMP) 2, transforming growth factor (TGF) β, CXCL1, CXCL2, CXCL6, vascular endothelial growth factor (VEGF), and Fibroblast Growth Factor-2 (FGF2) [107-112]. Several groups have reported that IDO and prostaglandin E2 (PGE2) are key molecules involved in immunosuppression mediated by MSC [113,114]. IDO is inducible by IFNγ and catalyzes the conversion from tryptophan to kynurenine. This depletion of tryptophan from the environment can significantly suppress T cell proliferation [113]. The synergistic effect of PGE2 is supposed to work through an increased induction of IDO production in MSC [115]. Galectin-1 is a protein that is released into supernatants by cultured MSC. This lectin can strongly inhibit T cell proliferation but leaves NK cells unaffected [116]. The soluble isoform of HLA-G5 is secreted by MSC, especially after contact with allospecific T cells. The soluble HLA-G5 has been shown to suppress T cell proliferation, NK cell-mediated cytotoxicity and IFNγ production and to induce expansion of regulatory T cells (Tregs) [117]. Maccario et al demonstrated that MSC mediate inhibition of alloantigen-induced dendritic cell (DC) 1 differentiation and preferentially activate Tregs [118]. A specific mechanism inhibiting cytolytic cells by reduced production and secretion of granzyme B in the presence of MSC was observed by Patel et al [110].

Immunosuppressive properties of MSC most probably also depend on environmental factors. Human and murine MSC have been shown to express toll-like receptors (TLRs) and the ligation of TLR3 and TLR4 by their respective natural ligands, double-stranded RNA and LPS, prevented the MSC from inhibiting T cell responses by the down-regulation of Jagged-1 expression on MSC [119-121].

### Cell contact-dependent interactions of MSC and immunocytes

In addition to the soluble factors, several cell surface molecules have also been described as contributing to lymphocyte suppression. A mechanism specifically suppressing NK cell functions has been shown by Spaggiari et al [115] revealing that downregulation of activating NK cell receptors NKp30 (CD337), NKp44 (CD336), and NKG2D (CD314) inhibits NK cell functions. In a different study they also demonstrated that activated NK cells can kill MSC. However, activated NK cells also produce IFNγ, which in turn induces up-regulation of HLA class I on MSC [122]. Binding of HLA molecules representing the ligands for inhibitory receptors on NK cells result in suppression of NK cell function.

Immunoglobulin-like transcript (ILT) 2 (CD85j) is an inhibitory receptor expressed on NK cells. ILT2 is specific for several HLA-I molecules but binds to HLA-G with a 3- to 4-fold higher affinity than to classical HLA-I molecules [123]. HLA-G is expressed by MSC and binding to ILT2 on NK cells has been shown to inhibit the polarization of NK-cell lytic granules and proper formation of the immunological synapse, intracellular calcium mobilization and IFN-γ polarized production of NK cells [124].

### The immunoprivilege of MSC

MSC have been reported to be immunoprivileged, meaning that they do not challenge a response of allogeneic immune cells [97]. The mechanisms of immunoprivilege are largely unknown but are most probably due to low expression of MHC I and MHC II as well as the immunosuppressive functions reviewed above, and suggest active self protection of MSC. Recently, however, it has been shown that the state of immunoprivilege is not stable. In vitro and in vivo data showed that cellular differentiation of MSC causes transition from an immunoprivileged to an immunogenic phenotype inducing cellular cytotoxicity or immune rejection [125]. IFN-γ has been shown to induce expression of MHC-I and to a lower extent also MHC-II, increasing the antigen presenting capacity and hence immunogenicity of MSC [126]. High-dose IFN-γ-treated MSC (500 U/mL) could activate T-cells and initiate proliferation of allogeneic T cells. Thus, after activation MSC can lose their immunoprivileged status. On the other hand, Polchert et al demonstrated in a mouse model that the treatment of MSC with IFN-γ (500 U/mL) improved the immunosuppressive effect in a GvHD model despite upregulation of MHC molecules [89]. Furthermore, neonatal and aged MSC exhibit considerable differences in their functional abilities. Lower immunogenicity and stronger immunosuppressive capacity makes neonatal MSC appear to be more viable for therapeutic approaches [127].

For the clinical use of MSC, B cells seem to be a particular target. Whereas T and NK cell functions are consistently found to be suppressed by MSC in many studies, there are some contradictory data on MSC-mediated effects on B cells. Thus, Deng et al found in lupus model mouse strain BXSB a reduction of B cell proliferation induced after LPS stimulation and a decrease of Ig production when co-cultured with BALB/c BM-MSC [128]. Moreover, in the human system Corcione et al described that B-cell proliferation was inhibited by BM-MSC. In addition, B cell differentiation was impaired as IgM, IgG, and IgA production was significantly reduced. These effects were mediated by MSC production of soluble factors, as assessed by transwell experiments [129]. In contrast, Rasmusson et al. demonstrated an increased proliferation and IgG production of B cells after co-culturing with BM-MSC. B cell modulation was mediated by soluble factors (e.g. IL-6) secreted by MSC when PBMC were used as responder cells [130]. However, purified B cell required cell-cell contact to get activated by MSC. These findings are corroborated by another study. Traggiai et al also observed MSC-mediated activation of defined B cell subsets [131]. They measured increased polyclonal proliferation and differentiation of naïve and transitional B cells into Ig-producing cells. The promoting effect mediated by MSC was in this study cell-cell contact dependent as confirmed in a transwell system. Comparable results were obtained when peripheral B cells from SLE patients were analysed. Proliferation and differentiation of patients' B cells as well as IgM and IgG production was supported by BM-MSC.

The discrepancies of the studies on MSC-mediated B cell immunomodulation are difficult to explain and may be due to differences in experimental conditions and kinetics. In any case, a potential therapeutic use of MSC for treating autoimmune diseases such as lupus erythematosus in order to suppress autoantibody producing B cells has to be strongly reconsidered until definite and reliably reproducible data on MSC B cell interactions are available.

Taken together, MSC-mediated immunosuppression is a multifaceted phenomenon based on several mechanisms. MSC differentially regulate immune responses by inhibiting the differentiation of dendritic cells, increasing the number of Tregs and suppressing numbers and functions of effector T cells and NK cells (Figure 3). This is achieved via iNOS, heme oxygenase-1, PGE2, IDO and various growth factors, such as IL-10 and TGFβ. Also, up-regulation of HLA class I on MSC and down-regulation of activating receptors on NK cells could lead to decreased NK cell cytotoxity and proliferation. Some of the mechanisms require direct cell-to-cell contact, whereas others are mediated via soluble factors. There is a species dependent variation of the mechanisms contributing to immunosuppression and finally, MSC from different sources from the same individual can differ in the molecular basis of their induced immunosuppression. Upon stimulation MSC might lose the immunoprivileged status, antagonizing their immunosuppressive capabilities. Future directions of immunity-related MSC research should focus on clarifying the exact mechanisms underlying MSC-mediated immunosuppression and sustained immunoprivileging in order to make the effective and safe therapeutic use of MSC more feasible.

## Effects of micro RNA in MSC

MSC within a primary culture can also exhibit different states of activation which can be related to the expression levels of certain micro RNAs (miR) including miR335 [132]. miR are small non-coding RNAs of about 20 to 22 nucleotides, which, upon sequence-specific binding to mRNAs, repress the translation of the corresponding proteins or induce a subsequent degradation of the miR/mRNA complexes.

A variety of different miR play an important role in regulating differentiation pathways and cell fate in MSC which recently has been reviewed by Guo et al. [133]. For example, osteogenic differentiation of MSC can be blocked by miR-125b, miR133, miR135 and miR206 which attenuate the expression of ERBB2, RUNX2, Smad5 and connexin-43, respectively. Likewise, expression of further specific miR are involved in the regulation of adipogenic and chondrogenic differentiation and pathways beyond the mesodermal lineage [133]. Moreover, miR are also involved in the regulation of replicative senescence and wound healing of MSC. Thus, miR which target distinct DNA-methyl transferases can promote senescence of MSC [133]. Although the molecular mechanisms of MSC senescence after a limited number of cell divisions are still poorly understood, cell fusion processes which are known for MSC or asymmetric cell divisions may also contribute to this phenomenon which enables the segregation of daughter cells committed to either senescence or retaining reproductive capacity in correspondence to the parental cells [134].

Furthermore, MSC can secrete micro vesicles which contain certain pre-microRNAs [135]. The released exosomes facilitate cell-to-cell communications and thus, can alter cell activities in target cells.

A proposed MSC model suggested that high miR-335 expression contributes to a potential non-activated (silenced) MSC auto-maintenance state, in contrast to low levels of miR-335 which produce an activated state leading to proliferation, migration and differentiation in MSC [132]. Of interest, a functional role in the regulation of epithelial-to-mesenchymal transition and a neoplastic development in breast tissue has also been attributed to miR-335 [136]. Moreover, MSC display some similarities to normal and tumorigenic human breast epithelial cells with respect to the gene expression pattern [137] and some surface receptor levels [138,139]. Whereas the location of MSC in the adipose tissue of the breast adjacent to mammary epithelial cells enables interactions by stimulatory cytokines and/or miR-containing micro vesicles, these stimulatory effects suggest a close functional relationship between these cell types [140]. Indeed, previous work has demonstrated that although MSC themselves do not develop teratoma even when derived from a teratoma-forming human embryonic stem cell line [141], a close vicinity to neoplastic breast epithelial cells within the tissue microenvironment can stimulate growth and metastasis of breast cancer cells by cytokines including CCL5 (Rantes) [142] and may most probably also influence the exchange of miR-containing micro vesicles. Thus, synergistic effects of MSC in cooperation with other cell types, e.g. tumor cells must be considered and require further elucidation.

## Concluding remarks

MSC represent an important stem cell population with multipotent capabilities which are extremely useful for clinical applications. Although certain discrepancies within the MSC literature result in differing descriptions of the biological properties of MSC, these effects may be explainable in part by the existence of distinct subpopulations within a tissue-derived primary culture that exhibit some variation in function [33,34,92]. Moreover, different isolation methods of MSC, particularly the use of proteases to digest the extracellular matrix for an enrichment of the stem cells, may alter MSC functions by non-specific degradation e.g. of certain surface receptors, whereas the explant culture of MSC from tissue pieces avoids such potential artifacts.

**Figure 4:**
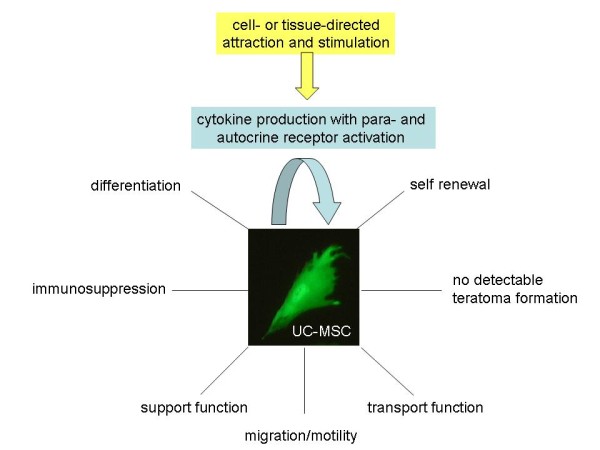
**Major characteristics of MSC and their functions *in vitro *and *in vivo ***. MSC are involved in important pathways of cell renewal by mediating the regeneration of damaged or aging cells, supporting tissue repair at wound sites and modulating immune functions. Due to the migratory capabilities, MSC can interact with and support damaged normal tissue cells in the local vicinity by providing an appropriate microenvironment.

In summary, MSC can self renew to a certain extend and differentiate (Figure 4). Moreover, they can display a variety of important cell functions in the organism including migration and transport functions to sites of local injuries or tissue damage to support appropriate cell and tissue renewal to replace the damaged areas (Figure 4). Concomitantly, MSC are non-immunogenic due to their immune-modulatory capabilities and no teratoma formation of MSC after allogenic human transplantations has been observed to date (Figure 4) which indicates an enormous potential for the clinical use of these cells, particularly in regenerative medicine.

Whereas a variety of different tissue sources for MSC have been described, MSC from birth-associated tissues, preferably parts of the placenta (i.e. D-MSC) and the umbilical cord/Wharton's jelly (UC- and WJ-MSC) may offer certain advantages. These include their non-invasive and ethically non-problematic availability. More importantly, MSC from these neonatal tissues possess increased proliferative capacity *in vitro*, especially under hypoxic conditions, in comparison to some MSC populations obtained from adult tissues. Quiescent stem cells within their niches of various tissues can be activated if required, however, even a reprogramming of cells via a retrodifferentiation program [143] or further processes to rejuvenate cells to a more juvenile and undifferentiated phenotype [134] are often not sufficient to cope with tissue requirements after injury or disease-associated tissue damage and degeneration. Therefore, in contrast to the limitations of bone marrow or adipose tissue, MSC from birth-associated tissues can be obtained in large quantities, and the required numbers of these stem cells can be transplanted in therapeutic approaches for tissue replacement.

Taken together, multifunctional MSC from parts of the placenta and the umbilical cord may represent a very promising stem cell population in regenerative medicine.

## Competing interests

The authors declare that they have no competing interests.

## Authors' contributions

CK and SB contributed the adult MSC part including Figure 2. RJ focused on the immune functions of MSC and provided Figure 3. RH contributed the other parts including the focus on embryonic/neonatal tissue-associated MSC, hypoxia and miRNA. Furthermore, RH provided Figures.1 and 4 and drafted the manuscript. All authors have read and approved the final version of the manuscript.
